# Single 0.9-mg intramuscular rhTSH dose achieves target TSH levels in differentiated thyroid carcinoma

**DOI:** 10.1007/s12020-026-04671-y

**Published:** 2026-06-02

**Authors:** Pedro Henrique Furst Leite, Magda Carvalho Pires, Gustavo Cancela Penna

**Affiliations:** 1 Department of Nuclear Medicine, Hospital Orizonti, Belo Horizonte, Brazil; 2 Department of Statistics, Institute of Exact Sciences, Federal University of Minas Gerais (UFMG), Belo Horizonte, Brazil; 3 Department of Internal Medicine, Federal University of Minas Gerais (UFMG), Belo Horizonte, Brazil

**Keywords:** Differentiated thyroid cancer, Radioactive iodine, Radioiodine therapy, rhTSH, Thyroid-stimulating hormone

## Abstract

**Background:**

Differentiated thyroid carcinoma (DTC) is the most common thyroid malignancy, with incidences increasing globally. Radioisotopic therapy with iodine-131 (RAI) is recommended after total thyroidectomy for patients at intermediate/high risk. A new approach that involves the use of recombinant human TSH (rhTSH) to stimulate iodine-131 uptake during RAI has long been approved. In the standard protocol, 0.9 mg of rhTSH is administered intramuscularly on two consecutive days, followed by a therapeutic dose of iodine-131 on day 3. Unlike the traditional method of levothyroxine (LT4) withdrawal, this protocol allows the continuation of LT4, avoiding the symptoms and metabolic consequences of profound hypothyroidism. However, its high cost remains an important limitation in our setting. Therefore, this study evaluated whether a single intramuscular dose (0.9 mg) of rhTSH, instead of the currently recommended two doses, is sufficient to achieve the target thyroid-stimulating hormone (TSH) level (> 30 mIU/L) currently recommended for RAI.

**Methods:**

This prospective, cross-sectional study included 88 patients. The TSH level on day 1 (TSH D1), defined as the primary outcome, was evaluated in relation to sex, age, body weight, recurrence risk (ATA 2025), baseline TSH values, and the interval between TSH measurements.

**Results:**

A single rhTSH dose substantially increased TSH levels to > 30 mIU/L in all patients. Age and body weight were independently associated with TSH D1.

**Conclusions:**

These preliminary results support the potential of single-dose rhTSH as a cost-effective and clinically feasible alternative in selected patients with DTC to achieve TSH levels > 30 mIU/L. Future comparative clinical trials should assess iodine uptake, therapeutic response, and long-term oncologic outcomes.

## Introduction

Following total thyroidectomy, radioiodine therapy (RAI) with iodine-131 may be indicated for ablative, adjuvant, or therapeutic purposes in patients with differentiated thyroid carcinoma (DTC), based on the AJCC/TNM 8th edition staging and the American Thyroid Association (ATA, 2025) recurrence risk criteria [1].

The efficacy of RAI rests on two pillars: a low-iodine diet and adequate elevation of thyroid-stimulating hormone (TSH) levels [1, 2]. This elevation upregulates expression of the sodium/iodide symporter (NIS), increasing radioiodine uptake by thyroid or neoplastic cells [3]. A serum TSH value considered adequate for this stimulation is > 30 mIU/L [4, 5].

Traditionally, TSH elevation was achieved by withdrawing levothyroxine (LT4), inducing a hypothyroid state; this remains the approach used in the Brazil’s public health system because rhTSH is inaccessible owing to its cost. Despite its efficacy and practicality, rhTSH has a major limitation: its high cost (approximately $1,000). However, this strategy carries undesirable clinical implications and negatively affects quality of life. As an alternative, recombinant human TSH (rhTSH), administered as two 0.9 mg intramuscular doses on consecutive days, has become the preferred strategy for most patients with DTC, as it effectively elevates TSH without requiring LT4 interruption [1, 2].

The two-dose regimen was established based on foundational preclinical and phase I/II studies [6–8] which, although demonstrating a biochemical response to a single dose, favored a second dose to ensure a more sustained TSH elevation. However, the clinical superiority of two doses over one has never been formally demonstrated in a large-scale clinical trial with oncologic endpoints.

In this context, we hypothesized that a single intramuscular dose of rhTSH could be sufficient to achieve TSH stimulation levels required for RAI.

This prospective study was designed to validate, in a contemporary cohort of patients with DTC, whether a single intramuscular 0.9 mg dose of rhTSH can raise serum TSH levels sufficiently above the recommended threshold of 30 mIU/L, providing a robust basis for the design of future dose de-escalation trials.

## Materials and methods

This prospective, cross-sectional study enrolled 88 patients with DTC who were referred for iodine-131 (131I) therapy under rhTSH stimulation, as recommended by their treating endocrinologists. Patient selection occurred between September 2022 and October 2024 at the Nuclear Medicine Department of Hospital Orizonti.

The study was approved by the Institutional Review Board of Hospital Orizonti and registered in the Plataforma Brasil national research ethics system (CEP/Conep) under protocol number CAAE 61747522.0.0000.0151. All participants provided written informed consent.

Clinical and laboratory data, including age, sex, body weight, date of total thyroidectomy, recurrence risk classification (ATA, 2025), administered 131I activity, baseline laboratory values (TSH, thyroglobulin, and anti-thyroglobulin antibodies), and TSH levels 22–24 h after the first rhTSH injection (TSH D1), were collected. RAI therapy was administered approximately 4 h after blood sampling for the TSH D1 assay, corresponding to a 26–28 h interval following the single rhTSH injection.

The inclusion criteria were: confirmed diagnosis of DTC, age ≥ 18 years, preparation for the first RAI dose with rhTSH, measurement of TSH levels 22–24 h after the first rhTSH dose, availability of complete clinical and laboratory data, and interval of < 90 days between baseline TSH and TSH D1 measurements.

The primary outcome, TSH D1, was analyzed in relation to sex, age, body weight, recurrence risk (ATA, 2025), baseline TSH levels, and the interval between baseline TSH and TSH D1 measurements.

### Statistical analysis

Quantitative variables were reported as the median and interquartile range (IQR), and categorical variables as absolute and relative frequencies (%). Statistical analyses were conducted using R software with a significance threshold of 5%.

TSH D1 was considered right-censored because of the laboratory assay’s upper quantification limit of 150 mIU/L. To account for this, a parametric regression model was fitted assuming a lognormal distribution for TSH D1. In this model, the logarithm of TSH D1 was expressed as a linear function of the following covariates: sex, age, body weight, recurrence risk category, baseline TSH level, and timing of TSH measurement,

Model coefficients were estimated using the partial maximum likelihood method, implemented via the survreg function in R software (version 4.5.0). To facilitate interpretation, exponentiated coefficients were reported as median ratios.

## Results

The final sample comprised 88 patients, 62.4% women, with a median age of 43 years (range, 18–77) and a median body weight of 75 kg (range, 50–139).

Regarding recurrence risk (ATA 2025), 12.5%, 28.4%, 39.8%, and 19.3% were of low, lower-intermediate, higher-intermediate, and high risk, respectively.

**Fig. 1:**
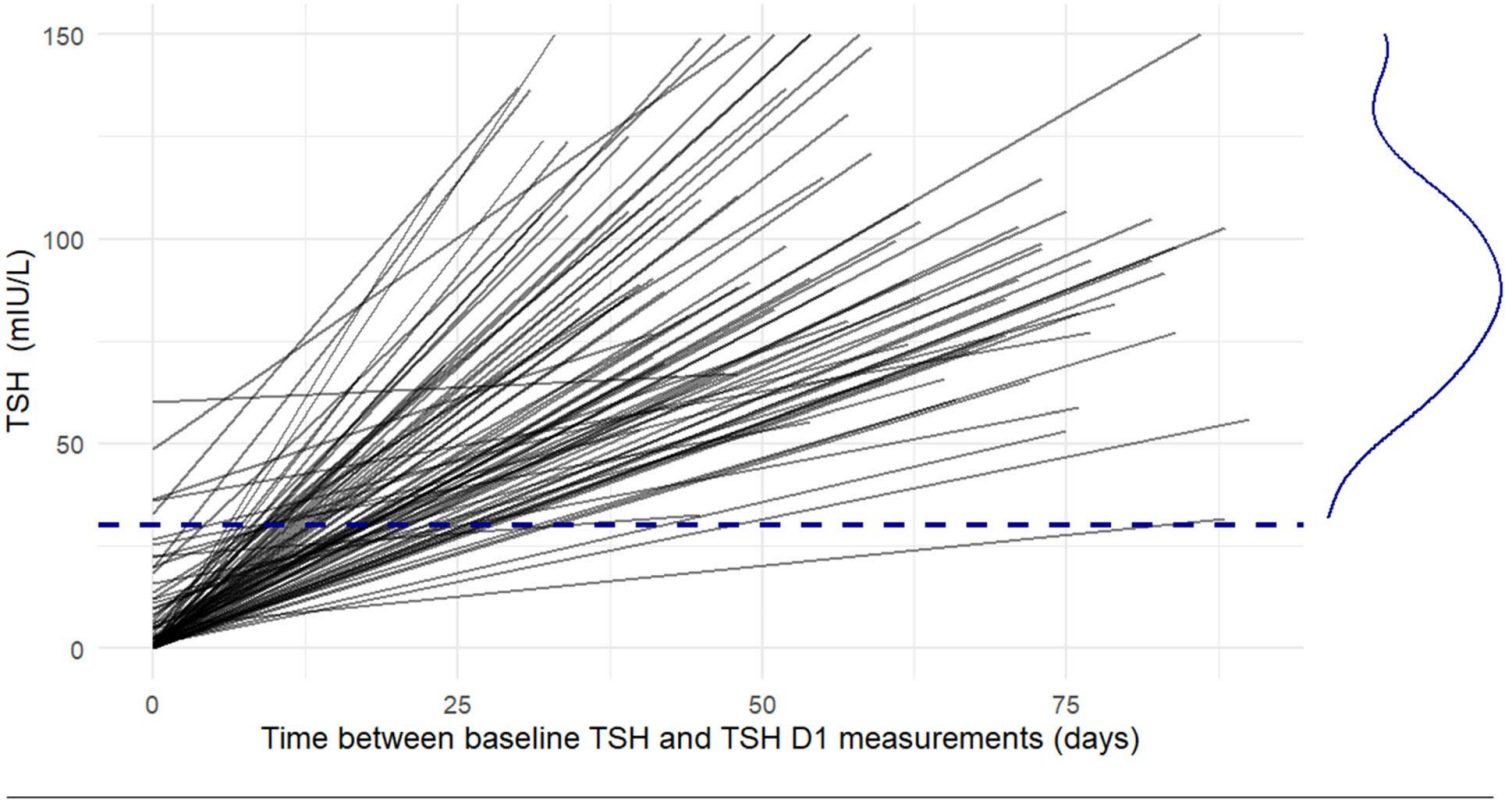
Individual patient responses to a single dose of rhTSH. Each gray line represents the TSH trajectory of a single patient over time. The dashed line indicates the 30 mlU/L target, achieved by all patients, The curve on the right illustrates the overall disptribution of TSH levels observed in the cohort

All patients (100%) achieved serum TSH D1 > 30 mIU/L, with a median level of 90.2 mIU/L (IQR = 73.81–110.23) (Fig. 1).

In the adjusted multivariate analysis, age and body weight were independently associated with TSH D1 (Table 1). Specifically, each 10-year increase in age was associated with a 6% increase in median TSH D1 (effect = 1.006; *p* = 0.010), and each 1-kg increase in body weight was associated with a 1.2% decrease in median TSH D1 (effect = 0.988; *p* < 0.001).

**Table 1:** Predictors of sufficient TSH response after a single rhTSH dose

	Coefficient	Effect (95 CI%)	*p*
Sex	0.000	1.000 (0.871–1.147)	0.994
Age	0.006	1.006 (1.001–1.01)	0.011
Weight	-0.012	0.988 (0.984–0.992)	< 0.001
RR low	-	-	-
RR low-intermediate	0.075	1.078 (0.887–1.311)	0.451
RR high-intermediate	0.004	1.004 (0.834–1.210)	0.964
RR high	-0.029	0.971 (0.789–1.196)	0.783
Time after TT	-0.001	0.999 (0.999-1.000)	0.136
Baseline TSH	0.017	1.017 (0.997–1.038)	0.088
Baseline TSH collection time	0.002	1.002 (0.998–1.005)	0.416
Interaction TSH	0.000	1.000 (0.999-1.000)	0.056

Effect = exp(coefficient); CI: confidence interval; RR: recurrence risk; TT: total thyroidectomy

## Discussion

In this context, administration of a single 0.9 mg dose of rhTSH was sufficient to raise serum TSH levels above 30 mIU/L in all patients with DTC, achieving a median of 90.2 mIU/L.

These results are consistent with evidence from early phase trials [8, 9]. It is worth noting that the currently approved two-dose rhTSH protocol was defined based on early preclinical and pharmacokinetic trials [6–8], which often used diagnostic rather than therapeutic endpoints, limiting their applicability to contemporary therapeutic contexts.

Although widely adopted, the conventional TSH target of > 30 mIU/L remains controversial [10, 11]. This threshold was initially established from observational data in the era of thyroid hormone withdrawal [5] and subsequently extrapolated to rhTSH protocols. Recent evidence has questioned the necessity and adequacy of this target, revealing a critical gap regarding the optimal peak level and duration of TSH elevation required to maximize therapeutic efficacy [10, 11].

This study also confirmed that body weight [12–14] and age [15–17] influence the response to rhTSH stimulation. The inverse relationship with body weight is likely explained by pharmacokinetic factors such as a larger volume of distribution [12], while the positive correlation with age may result from an age-related reduction in TSH metabolic clearance [15, 16]. Although all patients in this cohort achieved the target threshold, the negative correlation between body weight and serum TSH levels warrants vigilance for individuals with body weights significantly above the 75 kg median observed in our study. It would, therefore, be prudent for future, larger clinical trials to explore the establishment of a practical weight cutoff (e.g., 90–100 kg) or a weight-based dose adjustment. This would help ensure the universality and safety of the single-dose regimen, particularly in populations with a high prevalence of obesity.

Notably, our analysis did not find a significant association between sex and TSH response, in contrast with some previous findings [12, 17].

The limitations of this study include its modest sample size and single-center nature. Another limitation was the absence of data on renal function, a known predictor of peak TSH levels [15, 18]. Most importantly, as this study was not designed to assess clinical outcomes, it is crucial to highlight that a single dose might influence the kinetics of ¹³¹I differently compared to the standard dual-dose protocol, a point that warrants future investigation. A further limitation to be considered is the potential difference in NIS kinetics induced by a single-dose regimen compared to the standard two-dose protocol. Although our study demonstrates that a peak TSH > 30 mIU/L was consistently achieved, the duration of stimulation is inherently shorter. Functional NIS expression at the cell membrane is not an instantaneous process; it relies on a sustained TSH stimulus to ensure not only the initial iodide uptake (‘trapping’) but also its subsequent retention and organification. A shorter stimulation period could, theoretically, impact the effective half-life of radioiodine within remnant thyroid tissue or in metastases, a crucial determinant of the final absorbed radiation dose [19]. The literature suggests that iodine retention often follows a bi-exponential behavior, and optimizing the slow phase of this curve may depend on a more prolonged stimulus.

Nevertheless, its prospective design and the standardization of protocols are methodological strengths. These results may support the feasibility of prospective trials comparing one- versus two-dose rhTSH regimens and evaluating outcomes such as iodine uptake and long-term response.

If validated, a single-dose regimen could significantly reduce costs and improve patient convenience. This cost-reduction is particularly relevant in developing countries, where the standard two-dose protocol is often financially burdensome [20], aligning with the principles of value-based thyroid cancer care.

## Data Availability

No datasets were generated or analysed during the current study.
